# Correction: A novel implantable mechanism-based tendon transfer surgery for adult acquired flatfoot deformity: Evaluating feasibility in biomechanical simulation

**DOI:** 10.1371/journal.pone.0325521

**Published:** 2025-05-28

**Authors:** Hantao Ling, Ravi Balasubramanian

The Data Availability statement for this article is incorrect. The correct statement is: All OpenSim models, setup files, muscle activation files, data, and MATLAB scripts used to process the data are available from the Dryad data repository https://doi.org/10.5061/dryad.5hqbzkh4b.
