# Supplementary figures and images for: A novel implantable mechanism-based tendon transfer surgery for adult acquired flatfoot deformity: Evaluating feasibility in biomechanical simulation
Source: PLoS One. 2022 Sep 27;17(9):e0270638. doi: 10.1371/journal.pone.0270638 (PMC9514661; doi:10.1371/journal.pone.0270638)

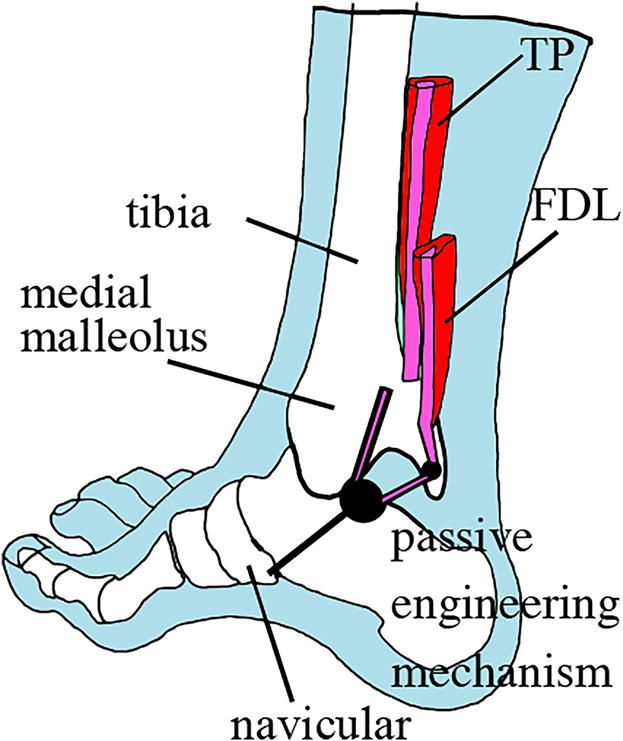

Supplement: S1 Fig — (JPG) [file pone.0270638.s001.jpg]
