# Supplementary material for: A novel implantable mechanism-based tendon transfer surgery for adult acquired flatfoot deformity: Evaluating feasibility in biomechanical simulation
Source: PLoS One. 2022 Sep 27;17(9):e0270638. doi: 10.1371/journal.pone.0270638 (PMC9514661; doi:10.1371/journal.pone.0270638)
Supplement: Attachment — Submitted filename: PONE-D-20-33947 - Review.pdf [file pone.0270638.s002.pdf]

PONE-D-20-33947 - Review

Ling, Hantao; Balasubramanian, Ravi

"A novel implantable mechanism-based tendon transfer surgery for adult acquired flatfoot deformity: validation using biomechanical simulation"

Summary:

The authors present an adapted rigid body model (OpenSim) of the human lower extremity in states representative of normal, flatfoot, and two reconstructed flatfoot states. They further elaborate on how the medial/lateral load distribution is changed between the model states under conditions simulating dynamic single-leg stance gait.

The manuscript addresses a question with scientific merit and is well written. However, much detail is necessary to understand how the model was constituted and what assumptions were made with regard to the mechanics simulated. Finally, the study is more appropriately presented as an exploration of a potential new device. It is not clear what if any quantification is made of model biofidelity relative to the in vivo case.

General Comments:

1. Model simplicity
    a. The OpenSim model is very simple and while segment geometry is visualized, it has no effect on joint function. This does not diminish potential model utility, but the predictive quality of the model should be contextualized relative to these simplifications
    b. The referenced OpenSim model is built to mimic normal lower extremity <u>normal</u> joint function. The appropriateness of using 'normal' joint and muscle definitions when simulating gross deformity should be included and contextualized
        i. STRUCTURE
            1. The model is modified to mimic a flat foot arch by manually displacing the medial column segment of the model. This displacement is maintained through a torsional spring. It is not clear how this affects foot mechanics and no comparison to in vivo response is given.
            2. More detail would help understanding how the path of the transferred 'FDL' was achieved. It seems that by using the existing PTT model elements and simply assigning it the force trajectory of the FDL, you have neglected any effects of routing. Is this correct? Why is this justified?
            3. Needs figure of model
                a. Why constrained to sagittal?

ii. TENDON
    1. the muscle curves are directly applied as force, which assumes that not only is the activation unchanged but that the structural deformity (with accompanying tendon rerouting & length changes) has no effect on the force generated at a given % of the stance phase. Is it reasonable / useful to use these ‘normal’ inputs for this deformed case? Please justify.

iii. MUSCLE
    1. The in vivo muscles have finite contraction lengths which are proportional to the number of sarcomeres in series in the muscle belly. While these can remodel to accommodate within limited ranges to effect new contractile lengths, the overall contraction length of the FDL is relatively constant. The pully mechanism incorporated does indeed have the effect of doubling force output… but it does so at the expense of halving the excursion of the tendon.
    2. It is not clear that this limitation is reflected in the model
    3. It is not clear that the full dorsiflexion → plantar flexion ROM would be achievable if the effective excursion of the tendon were halved

2. Much more detail is needed on the model. While some details can be obtained through the provided OpenSim references, much more is needed to explain <u>HOW</u> the model is used:
    a. For example, the boundary conditions assigned to the model and which parameters were driven, which were reported, which if any were used in the control of the platform (this is done in the referenced robotic cadaver work… was it similarly implemented in the model?), which joint degrees of freedom were allowed and at what locations, what are the stiffnesses and permitted ranges of those joints, etc.
    b. How are the inertial properties of the limb implemented and at what rate are the motions simulated?
        i. In particular, it is not clear how the axial force of the model was implemented during the simulation. The initial position seemed to be tuned by incrementally lowering the vertical plantar surface of the foot toward loading platform until the reaction force generated by the sphere/platform penetration was approximately equal to BW. After initial positioning though it is not clear what was done.
            1. Since it is referenced as a forward dynamics simulation, presumably there is a dynamic axial force and the vertical displacement is free to change in order to maintain equilibrium. Is this correct?
        ii. The characteristic bimodal shape of the GRF curve captured for living subjects is the result of deceleration of their body mass at heel strike and acceleration during toe off. Here the subject body mass doesn’t

change in time during stance phase and rate of loading is critical. Due to the significant technical challenges with control and instability many (all?) forward dynamics experimental and model simulations that I'm aware of are typically executed much slower than what is reflected in the GRF curves in Fig 8.

  c. The above comments (a,b) are relevant because the GRF is reported as an output and used as demonstration of the models' fidelity. However, it seems that this is perhaps a controlled input. *IF* that is the case, its agreement is simply verification of the inputs and should not be used as part of the objective scoring of the model.

3. Validation versus exploration
   a. The model provides useful insight into flatfoot function and the mechanics of how tendon transfer reconstructions may work
   b. However, it is not clear what reference human or cadaver data is presented and how the model biofidelity is scored relative to it.
      i. See above comments with regard to the use of GRF
      ii. Figure 9 shows the sensitivity of the model to incorporation of the TT and TT+I. However, the reference normal (Black & Black Dashed) lines are also from the simulation. It is not clear how claims of biofidelity are judged here since all comparisons are between states of the same model under review.
         1. The magnitude of changes (medial load shifted to lateral) should be discussed relative to those reported in the literature for human or cadaveric experiments. References should be expanded to include such prior work.

Specific Comments:

181-183 Exploring *all* the sensitivities of the model is perhaps out of scope. However, to say that *any* exploration of sensitivity is out of scope is not reasonable. The work may show the promise of the technique but claims that the model is 'validated' are overstated.

224-225 This is the first mention of the any soft-tissue element in the model. How was the footpad implemented? Were there any other soft tissues (e.g., skin, fat, muscle, etc.?) If the full mass of soft tissues were not incorporated, how were the limbs inertial properties approximated?

237-238 Additional detail regarding how contact sphere diameter was derived from studies of foot shape

344-345 See General Comment 2. Detail should be added to Methods to elaborate on this point.

Figure Comments

FIG3 Suggest elaborating/expanding this figure with detail about the segments, joints, and contact spheres.

FIG4 This figure spot may be better used as a place to show the specifics of the device and resulting tendon routing in the various model states

FIG5 Label the subplots B,C to reflect they are input kinematics

FIG8 Caption Denote that the black line is experimental data(?) (cadaver or human subject)

FIG# A simulation matrix would help in showing what was simulated and what model attributes were changed to represent a given state
