# Supplementary material for: A novel implantable mechanism-based tendon transfer surgery for adult acquired flatfoot deformity: Evaluating feasibility in biomechanical simulation
Source: PLoS One. 2022 Sep 27;17(9):e0270638. doi: 10.1371/journal.pone.0270638 (PMC9514661; doi:10.1371/journal.pone.0270638)
Supplement: Attachment — Submitted filename: Response to Reviewers.pdf [file pone.0270638.s003.pdf]

**Response to Reviewers**

31 March 2021

The authors sincerely thank the reviewers for their thorough and thoughtful reviews. We have addressed all the reviewer comments. The detailed comments and suggestions have strengthened the merit of our paper, and we believe it is significantly stronger because of your effort. The authors' response is below.

**Addressing General Changes:**

When reviewing the comments, we felt that the reviewers may have perceived that the paper claimed that our models and simulations *clinically validated* the proposed implant-based procedure and also the broad applicability of our methodology to other foot pathologies. Thus, some of the reviewer's comments were addressing the perceived overreach. We did not intend to overreach. Thus, in response, we have narrowed down the paper's scope and language to instead focus on how the models and simulations help to answer questions about initial biomechanical feasibility of the implant-based procedure. Such a biomechanical feasibility study is critical alongside any other validation methods. The following are the revisions to address this aspect:

- The manuscript title was changed to reflect that we are evaluating *feasibility* of the implant rather than *validating* the implant-based procedure.
- The abstract's language was revised to also reflect that we are determining initial feasibility of the novel implant rather than validating the proposed procedure.
- The language in the main text was revised to be focused on evaluating feasibility rather than proving validity in the following lines:
    - Lines 38-40
    - Lines 62-66
    - Lines 98-101
    - Lines 122-125
    - Lines 127-129
    - Lines 172-176
    - Lines 238-240
    - Lines 351-353
    - Lines 432-435
    - Lines 454-465
    - Lines 533-539
- The paper's limitations are also addressed in order to better frame the purpose of the simulations presented. For example:
    - Lines 161-167: We added discussion of the limitations of basing the four foot models off of the Gait2392 model in OpenSim, which is for a healthy subject.
    - Lines 258-262: We addressed that having low resolution input muscle force data was a limitation.
    - Lines 267-270: We mention that our simulations do not account for the compensatory muscle activations that are likely to occur with the development of adult acquired flatfoot deformity.
    - Lines 273-276: We made the assumption that we are evaluating the best-case surgical scenario for the traditional FDL tendon transfer, which enables us to neglect effects of tendon routing that would be present in actual surgery.

    - Lines 293-297: We discuss how the excursion trade-off for the implant is not fully explored in this simulation.
    - Lines 495-531: The entire Limitations subsection of the Discussion section was rewritten to focus on three major limitations of our simulations: (1) certain biological processes cannot be accurately simulated computationally in a biomechanics simulation, (2) biomechanical simulations require model simplification and assumptions, which limit the scope that results can be extrapolated to a clinical setting, and (3) further biomechanical simulation is required to properly analyze the biomechanics of surgical variations.
    - Lines 546-554: A summary of the limitations of the work is provided here.

**Addressing Reviewer 1 Comments (original comments bolded with author response in normal text):**

- **Lines 7-9: Assuming you are using Johnson and Strom classification, this should be cited. Also, it should be clarified that at stage 2 the deformity is still flexible enough to be corrected.**
    - Line 7: The citation for Johnson and Strom has been added.
    - Lines 7-9: This was clarified to state that the flatfoot condition becomes permanent in stage III PT tendon dysfunction. The indication for surgical intervention was also clarified for when conservative treatment failed for stage II PT tendon dysfunction.
- **Line 19: My understanding is that the osteotomy may also be required depending on how much flexibility there is in the deformity, in some cases it may not be a matter of being able to generate enough force.**
    - Lines 21-27: The indication for an osteotomy following FDL tendon transfer was clarified with additional references here. The two primary reasons for an osteotomy in this specific case are to correct the flatfoot deformity and to optimize the muscle routing of the transferred FDL.
- **Line 22: This paragraph could be clearer about what stage of development the proposed implant is at, from the following paragraph and the discussion it appears that it's only been tested in a cadaver arm?**
    - Line 39: The pulley implant is at an early stage of development (biomechanical simulation and initial cadaver foot experiment completed. Cadaver arm experiment for validating the pulley is imminent). This is now reflected in the text.
- **Line 33-35: Note here that the pulley also reduces the range of motion of the muscle, it's not simply doubling the force for free.**
    - Lines 46-50: You are correct. We have clarified this trade-off in the text.
- **Line 61: I'm not familiar with the term "foot's frontal axis"; I assume this refers to the sagittal plane of the foot?**
    - Line 83: The language in this sentence was changed to use accurate terminology. We apologize for this oversight.
- **Lines 138-141: Not clear what the degrees of freedom of the new joints are and how they interact with the other segments? More detail required here.**
    - Lines 182-186: We have included more description about the degrees of freedom of all four joints in the foot models and provided more detail for how the four segments of the foot models move relative to each other.
- **Line 149: What properties were used for the deltoid and spring ligaments?**

    - Lines 200-204: We did not use any deltoid or spring ligaments in the foot models presented in this paper to reduce computational complexity. We have changed the language here for clarity.
    - Line 204: Instead we simplified arch stiffness with a torsional stiffness of 1 Nm/degree about the arch joint.
- **Lines 184-186: Limitation is that there are likely compensatory changes in the activity of other muscles in FF conditions, these are not accounted for.**
    - Lines 491-495: This is indeed a limitation of our study that is not accounted for, which is now more clearly stated in the text. However, by keeping the muscle activations consistent between the arched foot and flatfoot models, we have effectively isolated the effects of loss of PT muscle function.
    - Lines 267-270: We do believe that since the same methodology was used across all four foot models that we are still able to make between-model comparisons of treatment effectiveness relative to the flatfoot models.
- **Line 200: Did you try non-linear interpolation for these? The true activation patterns are unlikely to be linear between points.**
    - Lines 258-262: We did not try non-linear interpolation. We chose to use this low-resolution set of muscle activations because it was paired and used with the kinematic data fed into our simulation that was originally from the Robotic Gait Simulator experiments. While these input activations were not smooth, the generated forces within OpenSim are smooth due to the muscle dynamics. There are no jerky movements resulting from these input forces.
    - Lines 262-264: While the piecewise linear application of muscle forces is indeed a limitation of our study, we still believe that between-model comparisons are still valid since all our foot models use the same inputs.
- **Line 214: The RGS is limited to this speed by physical constraints, did you consider increasing the speed of your simulation back to normal?**
    - Lines 302-307: We did consider raising the speed of the simulation, but ultimately chose to remain consistent with the data from the RGS experiment. We more clearly stated this in the text.
- **Line 221: Most simulations of the foot now use more complex models that can account for the non-linear viscoelastic behavior of the foot tissue.**
    - While it’s true that there are more complex foot models that account for non-linear viscoelastic behavior of the foot tissue, as far we know, these models are not dynamically simulated in gait to generate ground reaction forces. Instead, they may use ground reaction forces as an input to simulation rather than produce it as an output.
- **Line 225: Deformations are likely very different between sites on the foot.**
    - Lines 314-316: We agree with this comment. However, the variations in deformation across the foot are not easily found in literature. Of the additional studies that we reviewed, we mostly found reference to heel pad deformations (about 1cm) and applications of the same stiffness across the sole of foot models. These additional references are included in the manuscript.
- **Line 225-226: More details on this needed about this process. Was this a static simulation? This one of the key parts for tuning the models and how it was performed makes the validation data more or less externally convincing.**
    - Lines 338-345: The loading methodology was expanded on in the text. This was, indeed, a dynamic simulation of gait. The body weight load was applied in the model by

adjusting the distance between the foot model and ground plane at 0% stance phase. After setting this distance, contact and loading between the model and ground plane was maintained purely by the prescribed ground plane kinematics, muscle activations, and friction.

- **Line 229: Vs symbol different in the equation and definitions? And what value was used for Fv?**
  - Lines 319-320: The difference in v_s symbols was fixed.
  - Lines 315-318: The viscous friction was set to an arbitrary very large value because we assumed in our model that there would be no slip. This is now reflected in the text.
- **Line 247-248: How did the AP and ML reaction forces look?**
  - The AP forces that were generated had a similar shape profile and magnitude to those found in the literature. The ML forces were of similar magnitude but of differing shape profile. We ultimately decided not to include these forces as part of our analysis to prevent from detracting from the main point of our work, which is to demonstrate biomechanical feasibility of the implantable mechanism for shifting vertical ground reaction forces.
  - Please see ‘Addressing General Changes’ section above. We shifted the paper’s focus paper to be more on the novel implant rather than broadly validating our models for evaluating all types of ground reaction force reproduction.
- **Lines 280-290: I’m not sure how directly comparable measurements of plantar pressure are to the discrete contact spheres used in these models.**
  - Please see the ‘Addressing General Changes’ section above, where we state that we have narrowed the paper’s scope. We want to make clear that the paper’s intent is not to develop a full-proof simulation for precisely modeling the plantar distribution of forces across the entire sole of the foot during gait. Instead, we focus on the medial/lateral shift of the vertical ground reaction forces, which are comparable to plantar pressure data.
  - Lines 420-423: We have made also added additional comparison to shifting center of pressure data previously generated by the Robotic Gait Simulator.

**Addressing Reviewer 2 Comments (original comments bolded with author response in normal text):**

1. **Model simplicity**
   a. **The OpenSim model is very simple and while segment geometry is visualized, it has no effect on joint function. This does not diminish potential model utility, but the predictive quality of the model should be contextualized relative to these simplifications**
      i. The scope and claims of the predictive quality of the model has been narrowed throughout the manuscript according to the ‘Addressing General Sections’ above.
      ii. Lines 499-518: A new paragraph on model simplifications has been added to the manuscript to address these concerns.
   b. **The referenced OpenSim model is built to mimic normal lower extremity normal joint function. The appropriateness of using ‘normal’ joint and muscle definitions when simulating gross deformity should be included and contextualized**

i. Lines 161-167: We have included additional context about the effects of basing our flatfoot models on the Gait2392 model, which was created to model a healthy subject.

c. **Structure**

i. **The model is modified to mimic a flat foot arch by manually displacing the medial column segment of the model. This displacement is maintained through a torsional spring. It is not clear how this affects foot mechanics and no comparison to in vivo response is given.**

1. Lines 83-86: One of the purposes of the foot arch joint in this work was to enable simple adjustment between an arched foot and flatfoot model. This is now better described in these lines.
2. While we do not provide a direct analysis of how the foot arch affects foot mechanics, our distributions of vertical ground reaction force data indicate that the collapse of the foot arch in combination with the altered tendon routing and muscle activations are producing strongly relevant results.

ii. **More detail would help understanding how the path of the transferred 'FDL' was achieved. It seems that by using the existing PTT model elements and simply assigning it the force trajectory of the FDL, you have neglected any effects of routing. Is this correct? Why is this justified?**

1. Lines 273-276: We made the assumption that we are evaluating the best-case surgical scenario for the traditional FDL tendon transfer to best evaluate the theoretical maximum impact of our proposed implant. Here "best-case" refers to neglecting effects of tendon routing that would be present in actual surgery.
2. Lines 520-524: This was also expanded on as part of the limitations of our study and a direction for future study in biomechanical simulation.

iii. **Needs figure of model**

1. A new figure was generated (new Fig 4) of all four foot models and their major differences.
2. **Why constrained to sagittal?**
    a. Lines 229-231: The implant movement was constrained to the sagittal plane for simplicity. Specifically, the model evaluated the implant in ideal conditions without twisting or rotating. This is now described in the text.

d. **Tendon**

i. **The muscle curves are directly applied as force, which assumes that not only is the activation unchanged but that the structural deformity (with accompanying tendon rerouting & length changes) has no effect on the force generated at a given % of the stance phase. Is it reasonable / useful to use these 'normal' inputs for this deformed case? Please justify.**

1. The reviewer is correct. We have assumed that all muscle activations across the four models other than those of the FDL and PT are to remain constant.
2. Lines 280-286: We believe that this does not limit comparison of our results between our models. It in fact isolates the effects of removing PT

function in the flatfoot and transferring the FDL tendon in the treatment models.

e. **Muscle**
    i. **The in vivo muscles have finite contraction lengths which are proportional to the number of sarcomeres in series in the muscle belly. While these can remodel to accommodate within limited ranges to effect new contractile lengths, the overall contraction length of the FDL is relatively constant. The pully mechanism incorporated does indeed have the effect of doubling force output… but it does so at the expense of halving the excursion of the tendon. It is not clear that this limitation is reflected in the model.**
    ii. **It is not clear that the full dorsiflexion/plantar flexion ROM would be achievable if the effective excursion of the tendon were halved.**
        1. Lines 46-50: The excursion trade-off that is required for the force amplification provided by the implant is now included in the manuscript.
        2. Lines 293-297: Because our simulation kinematics are primarily driven by the moving ground plane, we are able to simulate the full dorsiflexion/plantarflexion range of motion of the stance phase of gait. We neglect the excursion trade-off in this simulation because our . We have addressed this limitation in the text.

2. **Much more detail is needed on the model. While some details can be obtained through the provided OpenSim references, much more is needed to explain HOW the model is used:**
    a. **For example, the boundary conditions assigned to the model and which parameters were driven, which were reported, which if any were used in the control of the platform (this is done in the referenced robotic cadaver work… was it similarly implemented in the model?), which joint degrees of freedom were allowed and at what locations, what are the stiffnesses and permitted ranges of those joints, etc.**
        i. Additional information was provided in the text to clarify what parameters were driving (inputs) vs. driven (outputs) for the simulation.
            1. Driving parameters: Muscle forces, ground kinematics
                a. Lines 287-293: Gait kinematics were expanded on.
                b. Lines 254-258: Muscle forces were expanded on.
            2. Driven parameters: Vertical ground reaction forces, joint kinematics
                a. Lines 310-315, 338-345: Vertical ground reaction forces were expanded on.
                b. Lines 343-345: Joint kinematics were expanded on.
    b. **How are the inertial properties of the limb implemented and at what rate are the motions simulated?**
        i. Lines 170-172: The inertial properties of the limbs were inherited from Gait2392 model, which used inertial properties derived from average anthropometric of 5 subjects. The reference is now included in the text.
        ii. **In particular, it is not clear how the axial force of the model was implemented during the simulation. The initial position seemed to be tuned by incrementally lowering the vertical plantar surface of the foot toward loading platform until the reaction force generated by the sphere/platform penetration was approximately equal to BW. After initial positioning though it is not clear what was done.**

1. **Since it is referenced as a forward dynamics simulation, presumably there is a dynamic axial force and the vertical displacement is free to change in order to maintain equilibrium. Is this correct?**
   a. Lines 338-345: The axial force was initially set by adjusting the distance between the foot model and the ground plane. After the initial distance was set, the axial load was purely determined by the ground plane kinematics, muscle forces, friction, and contact dynamics that were prescribed and set for the simulation.
   b. The axial force is not dynamically applied. Instead, the vertical ground reaction forces are dynamically generated as a result of the variables described directly above.
   c. The vertical displacement of the foot models never change since the models are all fixed in space at the tibia. The prescribed ground plane kinematics drive the displacement between the foot models and the ground plane.

iii. **The characteristic bimodal shape of the GRF curve captured for living subjects is the result of deceleration of their body mass at heel strike and acceleration during toe off. Here the subject body mass doesn't change in time during stance phase and rate of loading is critical. Due to the significant technical challenges with control and instability many (all?) forward dynamics experimental and model simulations that I'm aware of are typically executed much slower than what is reflected in the GRF curves in Fig 8.**

1. Lines 287-293: Since our foot models are fixed in space, we do not have to control the dynamics of accelerating a skeletal model. Instead, we prescribe a fixed kinematic trajectory to the ground plane and let the contact dynamics between the foot model and ground plane play out. Thus, we are capable of simulating at faster speeds because we avoid the complex control schemes typically required. This is the same methodology used with the Robotic Gait Simulator to use a single cadaveric lower limb specimen to simulate the stance phase of gait.

c. **The above comments (a,b) are relevant because the GRF is reported as an output and used as demonstration of the models' fidelity. However, it seems that this is perhaps a controlled input. *IF* that is the case, its agreement is simply verification of the inputs and should not be used as part of the objective scoring of the model.**
   i. Lines 76-79, 338-345, 469-471: To be clear, the GRF reported in this study are 100% generated by the simulation and not a controlled input. We have made this point clearer in the text.

3. **Validation versus exploration**
   a. **The model provides useful insight into flatfoot function and the mechanics of how tendon transfer reconstructions may work**
   b. **However, it is not clear what reference human or cadaver data is presented and how the model biofidelity is scored relative to it.**
      i. Lines 374, 392, 395, 423, 450: The referenced human/cadaver data are cited on these lines of the text. Some new references have been added for comparison.
      ii. **See above comments with regard to the use of GRF**

iii. **Figure 9 shows the sensitivity of the model to incorporation of the TT and TT+I. However, the reference normal (Black & Black Dashed) lines are also from the simulation. It is not clear how claims of biofidelity are judged here since all comparisons are between states of the same model under review.**
    1. Lines 499-518: A deeper discussion of how model simplification affects extrapolation to clinical settings as well as the reasoning for between-model comparison has been provided in the Limitations subsection of the Discussion. While direct clinical conclusions cannot be made, these models still provide a base for expected trends of the shifting of vertical ground reaction forces as a result of adult acquired flatfoot deformity and its surgical treatment options
    2. Figure 9 has also been edited with a smooth black line representing the arched foot vertical ground reaction forces for clarity.
    3. **The magnitude of changes (medial load shifted to lateral) should be discussed relative to those reported in the literature for human or cadaveric experiments. References should be expanded to include such prior work.**
        a. Lines 392, 395, 423, 450: More references have been included regarding medial/lateral shift of ground reaction forces, including those from the Robotic Gait Simulator experiment

4. **Specific Comments:**
    a. **181-183 Exploring *all* the sensitivities of the model is perhaps out of scope. However, to say that *any* exploration of sensitivity is out of scope is not reasonable. The work may show the promise of the technique but claims that the model is 'validated' are overstated.**
        i. The authors agree that claims made in the original manuscript about the broad applicability and validity of the models and simulations were overstated. We have changed the language of the manuscript to be more focused on exploring the feasibility of the proposed implantable device. See 'Addressing General Changes' section above for more detail. We would like the reviewer to know that the authors thoroughly explored different tendon configurations, contact dynamics parameters, and model simplifications in the developmental process.
        ii. Lines 236-240: We have also removed the original Lines 181-183 and instead replaced it with several parameters of the surgical procedure that could be analyzed in future studies with sensitivity analyses. Since the focus of this paper was on evaluating implant efficacy in the ideal scenario, we did not explore suboptimal surgical techniques here.
    b. **224-225 This is the first mention of the any soft-tissue element in the model. How was the footpad implemented? Were there any other soft tissues (e.g., skin, fat, muscle, etc.?). If the full mass of soft tissues were not incorporated, how were the limbs inertial properties approximated?**
        i. Lines 325-332: Additional detail was provided for how the footpad was implemented, including the contact spheres' placement and sizing.
            1. Line 315: And additional references for footpad deformation/stiffness.
        ii. Lines 345-346: No other soft tissue interactions were included in this work. This is now clearly stated in these lines.

iii. Lines 170-172: The inertial properties of the limbs were inherited from Gait2392 model, which used inertial properties derived from average anthropometric of 5 subjects. The reference is now included in the text.

c. **237-238 Additional detail regarding how contact sphere diameter was derived from studies of foot shape**

   i. Lines 325-332: We provided additional details on how the size and placement of the contact spheres were in these lines.

d. **344-345 See General Comment 2. Detail should be added to Methods to elaborate on this point.**

   i. Lines 466-477: The authors agree that more detail was needed for comparison to other foot model types in the literature. We have provided additional details as well as additional references.

5. **Figure Comments:**

   a. **FIG3 Suggest elaborating/expanding this figure with detail about the segments, joints, and contact spheres.**

      i. Since we have expanded on all of these aspects of the foot models in the text, we elected to keep the original figure as is.

   b. **FIG4 This figure spot may be better used as a place to show the specifics of the device and resulting tendon routing in the various model states**

      i. We have generated a completely new figure to demonstrate the mechanics of the pulley implant within the context of the biomechanical foot model (new Fig 5).

   c. **FIG5 Label the subplots B,C to reflect they are input kinematics**

      i. The subplots have been labeled to be clearer  that they are input kinematics.

   d. **FIG8 Caption Denote that the black line is experimental data(?) (cadaver or human subject)**

      i. The black line is not experimental data. It is the ground reaction forces generated by the arched foot model developed in this work.

   e. **FIG# A simulation matrix would help in showing what was simulated and what model attributes were changed to represent a given state**

      i. We did not generate this figure since we provided more detail within the text regarding simulation parameters.
