# Supplementary material for: A novel implantable mechanism-based tendon transfer surgery for adult acquired flatfoot deformity: Evaluating feasibility in biomechanical simulation
Source: PLoS One. 2022 Sep 27;17(9):e0270638. doi: 10.1371/journal.pone.0270638 (PMC9514661; doi:10.1371/journal.pone.0270638)
Supplement: Attachment — Submitted filename: Response to Reviewers.pdf [file pone.0270638.s005.pdf]

Response to Reviewers

08 June 2022

The authors sincerely thank the reviewers for their thoughtful reviews. We would like to thank Reviewer 1 for their diligence in this long process to strengthen our manuscript, and we would like to thank Reviewer 3 for stepping into the middle of the revision cycle to work out the final edits required to elevate this manuscript to the level of the journal. We have addressed all the comments laid out by the reviewers, and we believe our paper is much stronger because of your effort. The authors' response is below.

Addressing Reviewer 1 Comments (original comments bolded with author response in normal text):

1. **"The authors have addressed all of my comments in this much improved manuscript. Congratulations on an interesting study."**
    a. Thank you, again, for all your hard work over a long period of time on this work. We could not have done it without your efforts.

Addressing Reviewer 2 Comments (original comments bolded with author response in normal text):

1. **Both reviewers made comments that the intervention potentially reduces the range-of-motion (ROM) of the muscle (Reviewer 1) and the ankle joint (Reviewer 2). The authors revised and explained well why such an excursion trade-off is necessary to generate force that is 2X than what the FDL muscle is capable of. They also clarified that the full ROM from dorsiflexion to plantarflexion was simulated. The simulated ROM was passive (produced by the moving ground plate), while the previous reviewers' comments seem to concern the active ROM. The authors are recommended to discuss the potential effect or limitation on ROM to directly address the reviewers' comments.**
    a. It is true that the active range of motion of the foot is not explored in this study. However, in a previous cadaver study conducted by our collaborators, we showed that the implant-based procedure did not result in gait kinematics that exceed the physiological excursion limits of the FDL muscle. This is now reflected in the text in Lines 295-298.
2. **Reviewer 2 suggested to clarify inputs and outputs of the simulation and what reference human or cadaver data is presented. The authors revised by including additional references. Citation 35, 36, and 37 show that the RGS has been used to simulate physiological and pathological gait. By reviewing citation 11, the kinematics of the moving ground plate seems to come from the experimental data (output), which was generated by that tibia to ground kinematics of healthy participants and experimentally induced flat foot models (inputs). I understand it is difficult to classify whether the ground plate kinematics is physiological or pathological. What about the EMG estimates (line 252-254)? Do they come from healthy participants or not (line 252-254; citation 35, 42, 43)? If the data come from pathological population, what exactly are the pathologies?**
    a. The EMG estimates come from the healthy population and is more specifically detailed in the reference "Arthrodesis of the First Metatarsophalangeal Joint: A Robotic Cadaveric Study of Dorsiflexion Angle". We have revised a sentence in Lines 252-254 to clarify what population the EMG originates from and to include this reference. We simulated

pathology in the flatfoot condition by removing the activation of the PT muscle and replacing it with the FDL muscle properties and activations while removing the FDL muscle activation from its muscle. This is explained within the "Gait Simulation Kinetics and Kinematics" subsection.

3. **The same dimension of contact spheres were used for all four foot models. However, as suggested by citation 16 and 56, medio-lateral plantar pressure distribution can change before and after surgical intervention and between individuals presenting anatomical variations. This limitation is suggested to be discussed like the other model simplicity limitations (e.g., compensatory muscle activations).**
    a. We agree that this is a limitation of the study and our models. A sentence has been added in Lines 512-515 in the Limitations section to include this.
4. **The medial and lateral vertical ground reaction force in lines 385-395 do not completely match what are shown on figure 10. For example, in text, the arched foot showed medial and lateral 0.454 and 0.691 N/BW, respectively. However, on the figure, the respective medial and lateral peaks are about 0.4 and 0.6.**
    a. Thank you for the great catch! We mistakenly put the wrong values in the text. The figure values are correct, and those values are now reflected in the text in Lines 386-396.
5. **Line 46-50 regarding excursion trade-off may be moved and combined with lines 233-237.**
    a. We agree that this helps the flow of the paper. Lines 46-50 have been moved and combined with lines 233-237. The revised text segment is in Lines 231-235.
6. **Line 40-42 "…amplify the force transferred from the weaker FDL to the PT [tendon]"**
    a. Line 41: The authors agree that this is the appropriate verbiage for the sentence and have made the revision.
7. **Figure 8 in text shows gait events sequentially from left to right. It would be an improvement if a left lower leg is shown as compared to a right leg, allowing readers to visualize the foot proceeding the stance phase of gait from left to right. However, I understand that the authors might intend to show the medial side of the foot.**
    a. While the authors agree that the figure would help readers better visualize the progression of gait from the lateral side of the model, we have decided to keep the figure as-is for two reasons:
        i. The primary focus of the paper is on the posterior tibialis and flexor digitorum longus muscles and tendons and the medial longitudinal arch, which are all on the medial side of the model.
        ii. We considered mirroring the figure to maintain the view of the medial side. However, we don't want to confuse readers into thinking we used a left lower limb model when we state in the text that a right lower-limb model was used.
